# In vitro and ex vivo evaluation of preclinical models for FAP-targeted theranostics: differences and relevance for radiotracer evaluation

**DOI:** 10.1186/s13550-024-01191-6

**Published:** 2024-12-24

**Authors:** Circe D. van der Heide, Joana D. Campeiro, Eline A. M. Ruigrok, Lilian van den Brink, Shashikanth Ponnala, Shawn M. Hillier, Simone U. Dalm

**Affiliations:** 1 Department of Radiology & Nuclear Medicine, Erasmus MC University Medical Centre Rotterdam, Rotterdam, GD, 3015 The Netherlands; 2 Ratio Therapeutics, Inc., Boston, USA

**Keywords:** Cancer-associated fibroblast (CAF), Fibroblast activation protein (FAP), Targeted radionuclide therapy (TRT), Radionuclide theranostics, Preclinical models

## Abstract

**Background:**

Fibroblast activation protein (FAP) is an attractive target for cancer theranostics. Although FAP-targeted nuclear imaging demonstrated promising clinical results, only sub-optimal results are reported for targeted radionuclide therapy (TRT). Preclinical research is crucial in selecting promising FAP-targeted radiopharmaceuticals and for obtaining an increased understanding of factors essential for FAP-TRT improvement. FAP is mainly expressed by cancer-associated fibroblasts in the tumor stroma and less on cancer cells themselves. Therefore, other (complex) factors impact FAP-TRT efficacy compared to currently clinically applied TRT strategies. For accurate evaluation of these aspects, selection of a representative preclinical model is important. Currently mainly human cancer cell lines transduced to (over)express FAP are applied, lacking clinical representation. It is unclear how these and more physiological FAP-expressing models compare to each other, and whether/how the model influences the study outcome. We aimed to address this by comparing FAP tracer behavior in FAP-transduced HT1080-huFAP and HEK293-huFAP cells, and endogenous FAP-expressing U-87 MG cancer cells and PS-1 pancreatic stellate cells. [^111^In]In-FAPI-46 and a fluorescent FAP-targeted tracer (RTX-1370S) were used to compare tracer binding/uptake and localization in vitro and ex vivo. Additionally, FAP expression was determined with RT-qPCR and anti-FAP IHC.

**Results:**

Although FAP expression was highest in HEK293-huFAP cells and cell line derived xenografts, this did not result in the highest tracer uptake. [^111^In]In-FAPI-46 uptake was highest in HT1080-huFAP, closely followed by HEK293-huFAP, and a 6-10-fold lower uptake for U-87 MG and PS-1 cells. However, ex vivo U-87 MG xenografts only showed a 2-fold lower binding compared to HT1080-huFAP and HEK293-huFAP xenografts, mainly because the cell line attracts murine fibroblasts as demonstrated in our RT-qPCR and IHC studies.

**Conclusions:**

The interaction between FAP and FAP-targeted tracers differs between models, indicating the need for appropriate model selection and that comparing results across studies using different models is difficult.

**Supplementary Information:**

The online version contains supplementary material available at 10.1186/s13550-024-01191-6.

## Background

Fibroblast activation protein (FAP), a type-II transmembrane serine protease, is almost exclusively expressed by cancer-associated fibroblasts (CAFs) that are abundantly present in tumor stroma. High FAP expression is correlated with a poor outcome in multiple solid cancers, and together its prominent expression in > 90% of epithelial carcinomas and its tumor-promoting role, make FAP a promising target for anti-cancer interventions [1–3]. In line with this, many novel FAP-targeted radiotracers have been developed for targeted radionuclide imaging and targeted radionuclide therapy (TRT) in recent years [4]. For example, the successful FAPI series developed at the University of Heidelberg [5–7], of which [^68^Ga]Ga-FAPI-04 and [^68^Ga]Ga-FAPI-46 demonstrated potent lesion detection of more than 28 cancer types in clinical case studies [7, 8]. Next to these compounds, other novel FAP tracers have also shown promising imaging results in preclinical and clinical case studies [4, 9], and currently, multiple clinical trials for FAP-targeted theranostics are ongoing in various cancer types (e.g., NCT04849247, NCT05262855, NCT04939610, NCT04621435). Despite these promising developments and an accelerated development of many FAP-targeted radiotracers, only little therapeutic benefit has been demonstrated with FAP-TRT to date, mainly due to suboptimal tumor retention [6, 10], and future studies are needed to determine the anti-cancer potential of FAP-TRT.

With FAP-TRT the cancer cells are generally targeted indirectly via the FAP-expressing CAFs, in contrast to currently approved TRT strategies (e.g., targeting somatostatin receptor subtype 2) [11]. As a result, the cancer cells are exposed to cytotoxic radiation via crossfire effect only. Thus, the amount of damage inflicted to the cancer cell heavily depends on the FAP-expression level by the CAFs, the spatial location of the CAFs, and the penetration depth of the selected radionuclide [12]. Another aspect that could affect FAP-TRT efficacy is the interaction of the radiotracer with FAP, followed by its cellular processing. This interaction and processing are likely different for FAP expressed by CAFs, compared to targets expressed by genetically unstable cancer cells. Preclinical research is crucial to help determine the relevance of these complex aspects for cellular retention of the FAP-targeted radiotracers and the success of FAP-TRT.

To ensure accurate preclinical evaluation of FAP-targeted radiotracers, it is important to select appropriate models. Currently, FAP-transduced cancer cell lines are the most commonly used model for both in vitro and in vivo evaluation of FAP-targeted radiotracers, in particular the human fibrosarcoma cell line HT1080 and the human embryonic kidney cell line HEK293 [6, 7, 13–15]. Evidently, overexpression of FAP on the tumor cells does not represent the clinical tumor histopathology of FAP-expressing CAFs, situated next to the cancer cells [15]. Another model often used for in vivo studies is the human glioma U-87 MG cell line derived xenograft (CDX) model. U-87 MG cells have low FAP expression in vitro, but in vivo these cells can recruit FAP-positive murine fibroblasts to the tumor site [16–18], mimicking the patient histopathology somewhat more closely. It is currently unclear how these commonly used preclinical models compare to each other in terms of the interaction between FAP and the radiotracer, including its cellular processing, and how this relates to radiotracer interaction and behavior in natural FAP-expressing human CAFs. Accordingly, we aimed to compare the three aforementioned preclinical models, and included an endogenously FAP-expressing pancreatic stellate cell line (PS-1), in preclinical studies using the well-known FAPI-46 [19] labeled with indium-111 and a fluorescent FAP-targeting small molecule also based on UAMC-1110, coupled to an Alexa Fluor™ 568 dye (RTX-1370S). We compared the models in assays commonly used for assessing FAP-targeted radionuclide theranostics, to evaluate whether and how the model influences the outcome.

## Materials & methods

### Cell culture

Experiments were performed using five human cell lines. The fibrosarcoma cell line HT1080, stably transduced with human FAP (huFAP) as previously described [20], was kindly provided by Dr. Uwe Haberkorn (University Hospital of Heidelberg), and wild-type HT1080 (HT1080-WT) was purchased (ATCC). Human Embryonic Kidney cells (HEK293), overexpressing huFAP fused with green fluorescent protein (GFP) after stable transduction, were transduced according to a protocol described in the supplementary information. PS-1 pancreatic stellate cells, isolated from the pancreas and immortalized with telomerase reverse transcriptase (hTERT) [19], were provided by Queen Mary University (London). The glioblastoma cell line U-87 MG (Merck Millipore, Darmstadt, Germany) was purchased. All cell lines were cultured in DMEM™ Glutamax, supplemented with 10% fetal bovine serum (Gibco, Breda, The Netherlands) and 100 UI/mL penicillin and 100 µg/mL streptomycin (Merck Life Science NV, Amsterdam, The Netherlands). The cells were maintained at 37 °C/5% CO_2_ and routinely passaged. For all assays, cells were seeded one day prior to the experiment to reach 80% confluence. For confocal microscopy studies, cells were seeded on 18 mm #1.5 coverslips (Marienfeld, Lauda-Königshofen, Germany).

### Radiolabeling

FAPI-46 (MedChemTronica, Sollentuna, Sweden) was radiolabeled with [^111^In]InCl_3_ (Curium Pharma, Petten, The Netherlands) with a molar activity of 20 MBq/nmol for all studies, as previously described [21]. The incorporation of indium-111 was measured using instant thin-layer chromatography and radio-high-performance liquid chromatography (Fig. S1). For all studies the radiochemical yield of [^111^In]In-FAPI-46 was > 95%.

### Radiotracer internalization studies

One day after seeding, the cells were washed twice with warm phosphate-buffered saline (PBS) (Gibco) and incubated at 37 °C or 4 °C for 5–180 min with 0.5 mL incubation medium (DMEM™ Glutamax, 1% bovine serum albumin (BSA), 20 mM HEPES (pH 7.4)) containing 1 nM [^111^In]In-FAPI-46, +/- 1 mM UAMC-1110 (Biosynth Ltd, Berkshire, United Kingdom) to determine specificity of uptake. After incubation, the medium was removed and cells were washed twice with cold PBS. Subsequently, cells were lysed using 1 M NaOH for 20 min at room temperature (RT) to measure total [^111^In]In-FAPI-46 uptake. When indicated, cells were incubated with 50 mM Glycine–100 mM NaCl acid buffer (pH 2.8) for 10 min at 4 °C prior to the NaOH incubation, to separate the membrane-bound and internalized fraction of [^111^In]In-FAPI-46. Hereafter, the collected fractions and standards of 100 µL radioactive incubation medium, were measured in a γ-counter (HIDEX, Turku, Finland). The data are expressed as the percentage of added activity per 200,000 cells (% AA/200,000 cells), as this reflects the average of the cell counts across all four cell lines.

### Chemistry

The fluorescent FAP-targeted small molecule RTX-1370S, was obtained via multistep syntheses as described in the supplementary material and as depicted in Scheme 1 (Supplementary Materials). In short, compound **3** was obtained by a peptide coupling reaction of commercially available **1** and **2**. The Fmoc group in **3** was removed using diethylamine in THF solvent at room temperature to yield **4**, which was then coupled with compound **5** using HATU reagent to give **6**. Compound **6** underwent a deFmoc reaction to produce key Intermediate-**A**. Another key Intermediate**-B** was synthesized using the previously reported synthesis [22]. Both Intermediates **A** and **B** were coupled to give **13** in good yields, which was further undergo de-Boc reaction formed **14**. In the final step, the compound **14** was coupled with Alexa Fluor™ 568 produced RTX-1370S. The purity of the final compound wasdetermined by liquid chromatography–mass spectrometry was 98.6% (Fig. S2).

### Confocal microscopy

Fluorescent tracer uptake was determined using RTX-1370S. Cells grown on coverslips were incubated with cell culture medium containing 10 nM RTX-1370S for 15 or 45 min at 37 °C or 4 °C. Following incubation, cells were fixated using 2% paraformaldehyde and mounted with Vectashield^®^ Antifade Mounting Medium containing DAPI (Vector Laboratories, Newark, CA, USA). Data acquisition was performed with a Leica TCS SP8 confocal microscope (Leica Microsystems, Wetzlar, Germany), using the 40x objective. The parameters used were λ_EX_ 405 nm/λ_EM_ 420–470 nm for DAPI, λ_EX_ 488 nm/λ_EM_ 520–560 nm for GFP, and λ_EX_ 561 nm/λ_EM_ 590–620 nm for RTX-1370S. All images were acquired with the same microscope settings and analyses were performed using LAS X Office software (Leica Microsystems) and Fiji [23]. Co-localization was analyzed using the JacoP plugin for ImageJ software (version 1.54f) based on Mander’s coefficient.

### Collection of cell line derived xenograft material for ex vivo analyses

Details on the development and collection of CDX material from HEK293-huFAP and U-87 MG are described in the supplementary material. HT1080-huFAP and the non-FAP-expressing HT1080-WT CDX tumors were obtained in our lab as described previously [21].

### Immunohistochemistry of cell lines and xenograft material

Cytospins of the cell lines, obtained by centrifuging 100 µL cell suspension containing 50,000-100,000 cells for 6 min at 1200 RPM (Rotofix 32 A, Hettich, Geldermalsen, The Netherlands), were fixated with 10% buffered formalin for 1 h. Thin sections (4 μm) of formalin-fixed paraffin-embedded CDXs were sliced and subjected to standard hematoxylin and eosin (H&E) staining and anti-FAP DAB staining using the anti-FAP antibody EPR20021 (Abcam, Cambrdige, UK) in 1/50 dilution, with affinity for both human and murine FAP (muFAP). Detailed information on the immunohistochemistry (IHC) can be found in the supplementary material.

### Reverse transcription quantitative polymerase chain reaction of cell lines and xenograft material

To obtain material for the reverse transcription quantitative polymerase chain reaction (RT-qPCR) TRIzol^®^ reagent (Invitrogen, Carlsbad CA, USA) was used. Detailed information of the RNA isolation can be found in the supplementary material. After isolation, RNA concentration and purity (260/280 nm and 260/230 nm) were determined using the Nanodrop 2000 spectrophotometer (Thermo Scientific, Wilmington DE, USA), and 500 ng of the isolated RNA was reverse transcribed into cDNA using the RevertAid First Strand cDNA Synthesis Kit (Thermo Scientific). For the RT-qPCR a master mix was prepared containing 1 µL cDNA, 1.1 µL RNAse free H_2_0, 2.5 µL 2x SensiFAST SYBR^®^ Green Reaction Mix (Meridian Bioscience, Cincinnati, USA), 0.2 µL (10 µM) of both the forward and reverse primer, per sample and run on a QuantStudio™ 7 Flex Real-Time PCR system (Thermo Scientific). SYBR^®^ Green primer sequences used for RT-qPCR are described in Supplementary Table 1 (Life Technologies, Darmstadt, Germany), and primer specificity was confirmed by melting curve analyses. Data analyses was performed using the QuantStudio™ Real-Time PCR software V1.7.2, and results were used to determine the relative expression of huFAP and muFAP in relation to the geometric mean of housekeeping genes. Efficiency and relative quantification of the FAP expression was performed by the Pfaffl method [24].

### In vitro autoradiography on xenograft material

Fresh frozen tumors of HT1080-huFAP, HEK293-huFAP, U-87 MG, and HT1080-WT were sectioned into 5–10 μm slices and mounted on Starfrost microscope slides (Thermo Scientific). The autoradiography was performed as described previously [25]. In short, tissue slices were incubated for 1 h at RT with radioactive incubation buffer, containing 1% BSA and 1 nM [^111^In]In-FAPI-46 +/- 1 mM UAMC-1110 to determine the specificity of radiotracer binding. Following incubation, slides were washed 3 times and loaded into the BeaQuant system (Atlantic Instruments for Research, Nantes, France). Analyses were performed using the Beamage software version 3.1.1. (Atlantic Instruments for Research). Radiotracer binding was expressed as % AA measured in counts/mm^2^/min, and corrected for non-specific binding.

### Statistics

Data are expressed as means ± SD for at least three separate experiments. Statistical analyses were performed with GraphPad Prism version 9.0.0 (San Diego, California USA). Significance of the resultswas determined using an unpaired multiple t-test with a single pooled variance.

## Results

### [^111^In]In-FAPI-46 uptake over time

**Fig. 1:**
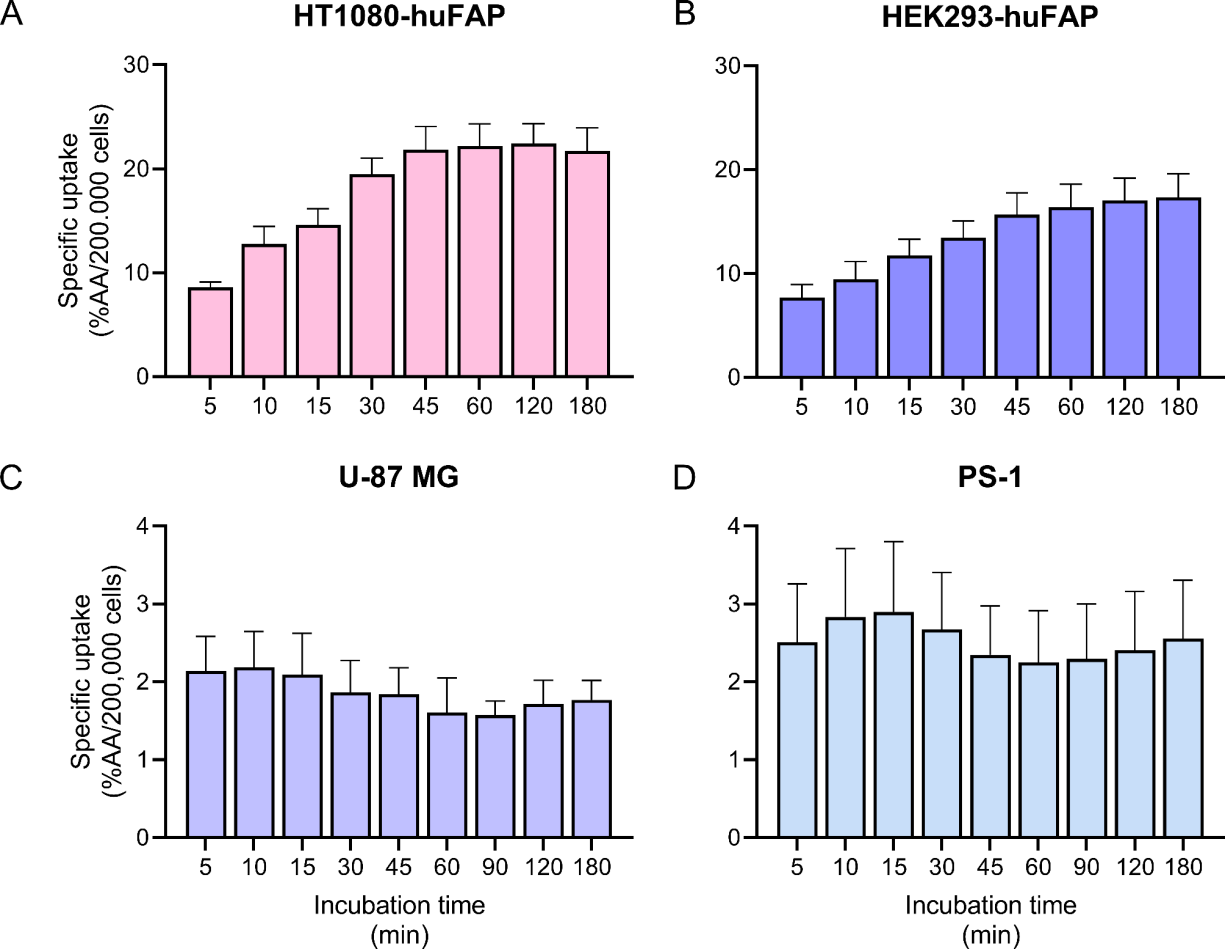
[^111^In]In-FAPI-46 uptake over time. Total uptake of 1 nM [^111^In]In-FAPI-46 by (**A**) HT1080-huFAP, (**B**) HEK293-huFAP, (**C**) U-87 MG, and (**D**) PS-1 cells after 5–180 min incubation

The total uptake of [^111^In]In-FAPI-46 by HT1080-huFAP cells increased over time, and reached a plateau between 45 and 120 min incubation, with the highest uptake measured at 120 min (22.4 ± 1.9% AA/200,000 cells) (Fig. 1A). A similar pattern was observed for HEK293-huFAP cells, although the [^111^In]In-FAPI-46 uptake reached its absolute peak after 180 min (17.3 ± 2.3% AA/200,000 cells) (Fig. 1B). Both the PS-1 and U-87 MG demonstrated a notably lower [^111^In]In-FAPI-46 uptake compared to HT1080-huFAP and HEK293-huFAP. Moreover, peak uptake was reached more rapidly, which was 2.2 ± 0.4% AA/200,000 cells at 10 min and 2.9 ± 0.9% AA/200,000 cells at 15 min for U-87 MG and PS-1, respectively (Fig. 1C, D).

### [^111^In]In-FAPI-46 uptake at 37 °C and 4 °C

**Fig. 2:**
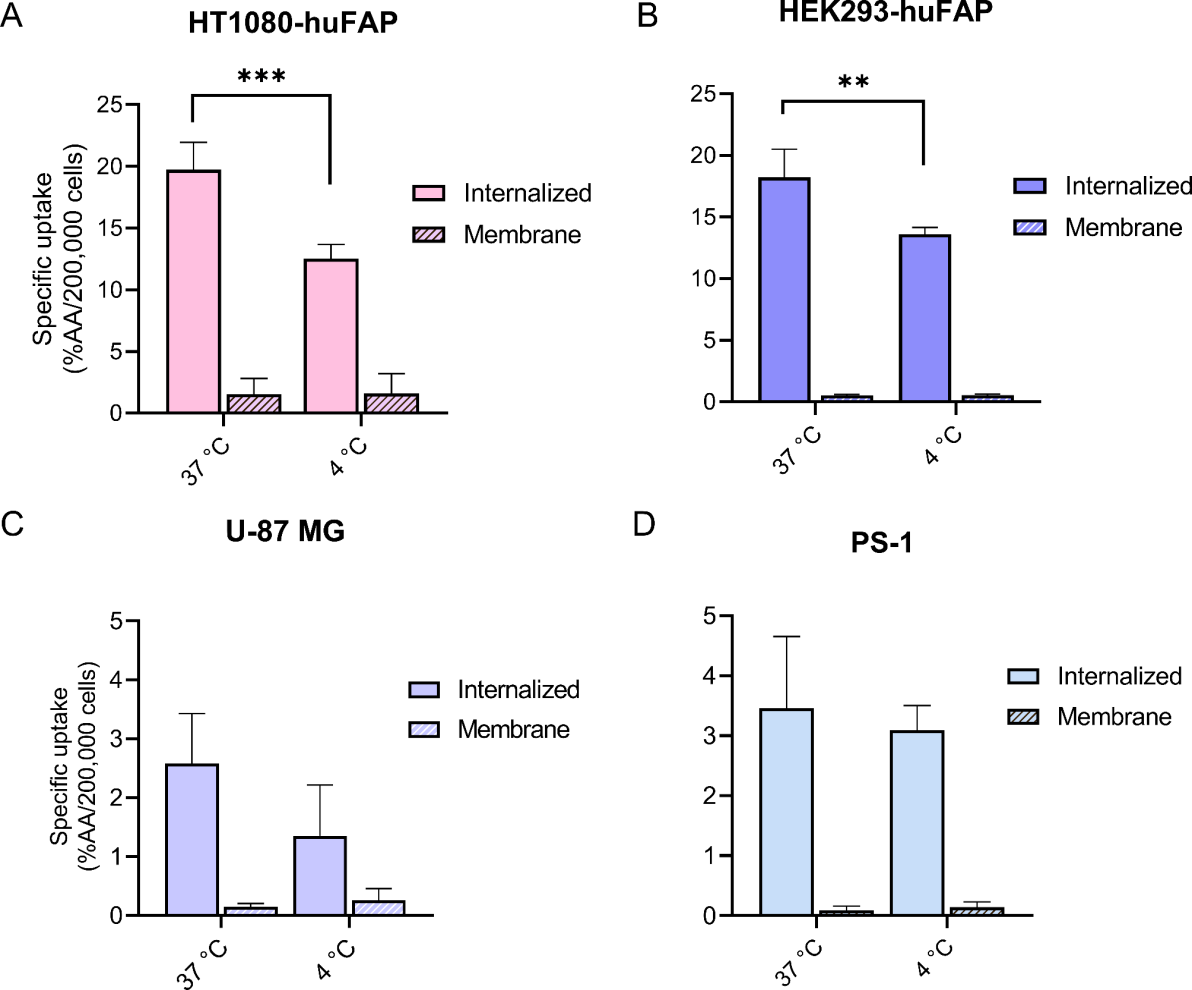
[^111^In]In-FAPI-46 uptake at 37 °C and 4 °C. Membrane-bound and internalized [^111I^n]In-FAPI-46 fractions separated after 45 min incubation. (**A**) HT1080-huFAP, (**B**) HEK293-huFAP, (**C**) U-87 MG, and (**D**) PS-1 cells (**p < 0.01, ***p < 0.001)

At 37 °C only limited membrane-bound [^111^In]In-FAPI-46 was detected in all cell lines, although slightly higher for HT1080-FAP cells than for HEK293-huFAP, U-87 MG, and PS-1 cells (1.6 ± 1.3 vs. 0.5 ± 0.08, 0.1 ± 0.06, and 0.09 ± 0.07% AA/200,000 cells, respectively) (Fig. 2). The membrane-bound [^111^In]In-FAPI-46 fractions were not significantly affected by incubation at 4 °C. In contrast, a significantly lower internalized fraction was observed at 4 °C compared to 37 °C for HT1080-huFAP cells (12.5 ± 1.2 vs. 19.7 ± 2.2% AA/200,000 cells, respectively *p* < 0.001) and HEK293-huFAP cells (13.6 ± 0.6 vs. 18.2 ± 2.3% AA/200,000 cells, respectively, *p* < 0.01), while the uptake was not significantly affected by the temperature for U-87 MG and PS-1 cells (Fig. 2).

### RTX-1370S localization

**Fig. 3:**
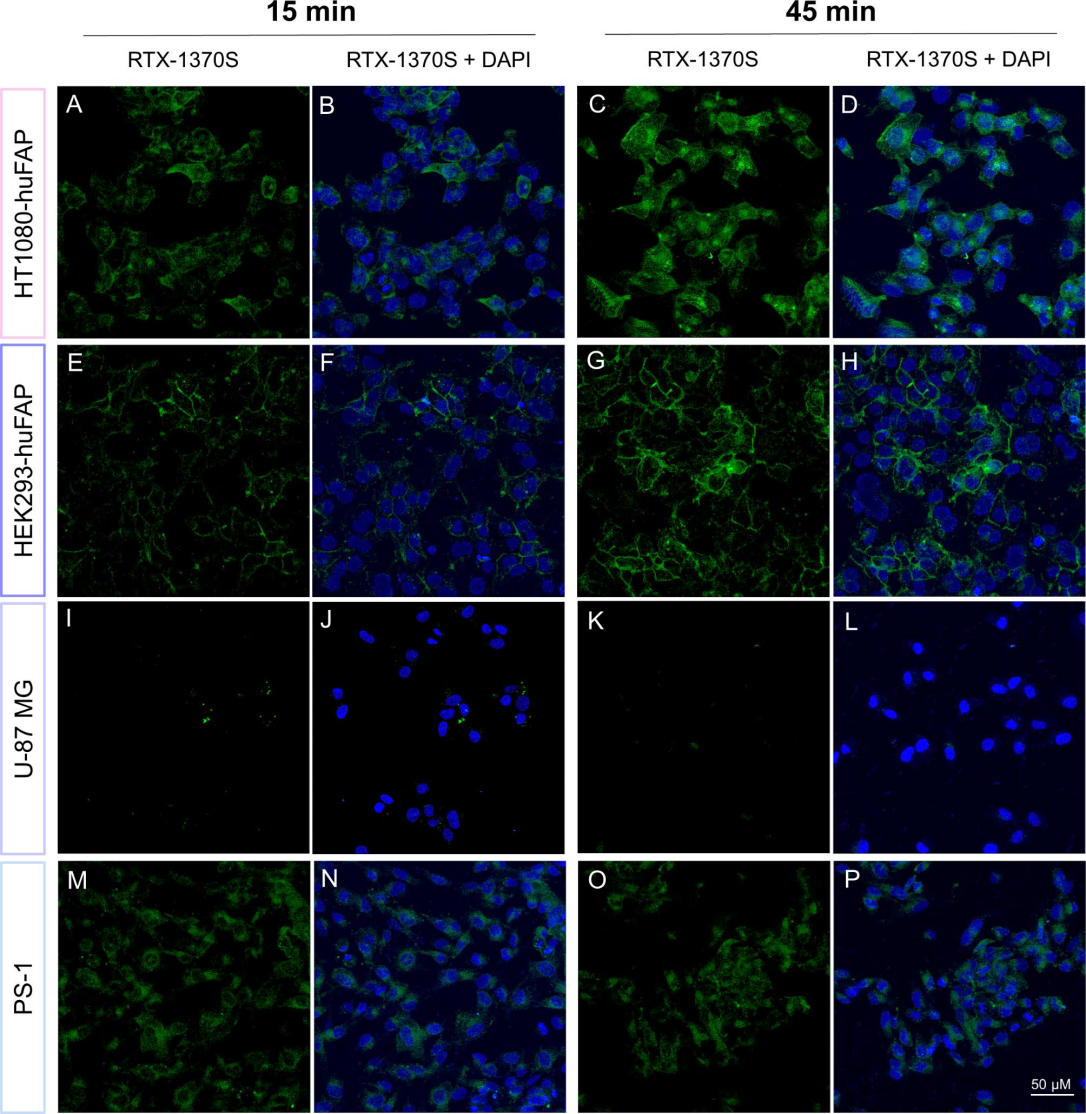
Confocal images of RTX-1370S uptake. Representative confocal microscopy images of the cells fixed after 15–45 min incubation with 10 nM RTX-1370S at 37 °C. On (**A**-**D**) HT1080-huFAP, (**E**-**H**) HEK293-huFAP, (**I**-**L**) U-87 MG, and (**M**-**P**) PS-1 cells. RTX-1370S was conjugated to an Alexa Fluor™ 568 (green) and nuclei were stained with DAPI (blue). Scale is equal for all panels

Uptake studies with RTX-1370S showed a distinct uptake pattern among the four cell lines. Similar to the results observed for [^111^In]In-FAPI-46, RTX-1370S showed the highest uptake in the two FAP-transduced cell lines (Fig. 3). However, in contrast to radiotracer uptake, RTX-1370S confocal microscopy demonstrated differences in the localization of the fluorescent tracer. In HT1080-huFAP cells, the Alexa Fluor™ 568 signal is visible both at the membrane and in the cytoplasm, and seems to accumulate near the nucleus, presumably in the perinuclear area (Fig. 3A-D). In contrast, in the FAP-GFP expressing HEK293-huFAP cells, RTX-1370S mainly accumulates on the cell membrane with less tracer observed in the cytoplasm (Fig. 3E-H). Additionally, RTX-1370S co-localization with the GFP signal was 82.9% and GFP localization with RTX-1370S somewhat lower with 72.6% (Fig. S3). Furthermore, although uptake of the fluorescent tracer in HT1080-huFAP and HEK293-huFAP was rapid, with a clear signal already after 15 min of incubation, a further increase in signal intensity was observed after 45 min incubation.

Almost no RTX-1370S uptake was detected in U-87 MG after either 15- or 45-min incubation with the fluorescent tracer (Fig. 3I-L). Although a much lower intensity was visible in PS-1 cells compared to the two transduced cell lines, clear internalization of RTX-1370S was observed (Fig. 3M-P).

**Fig. 4:**
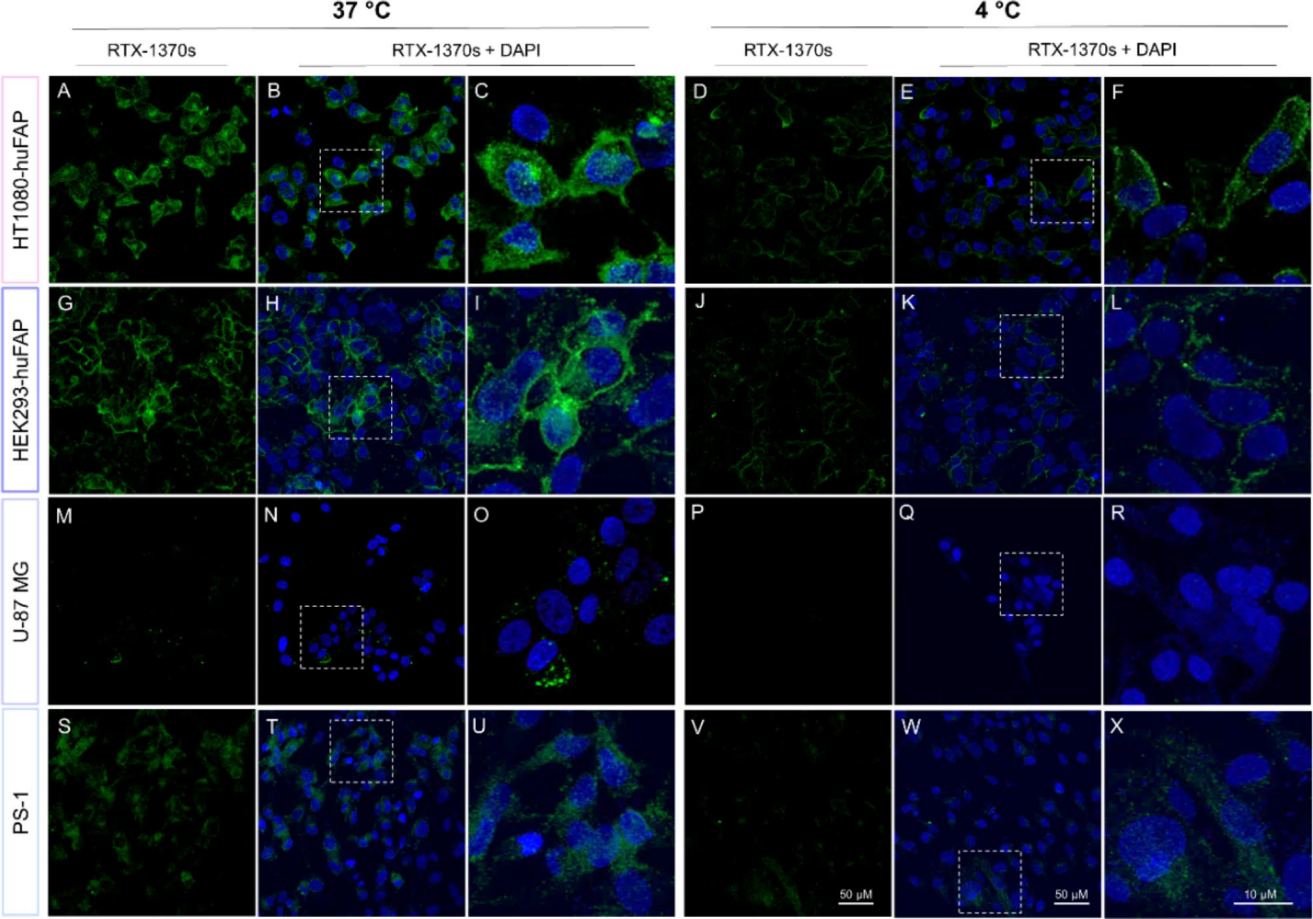
RTX-1370S uptake at 37 °C and 4 °C. Representative confocal microscopy images of the cells fixed after 45 min incubation with 10 nM RTX-1370S at 4 °C or 37 °C on (**A**-**F**) HT1080-huFAP, (**G**-**L**) HEK293-huFAP, (**M**-**R**) U-87 MG, and (**S**-**X**) PS-1 cells. RTX-1370S was conjugated to Alexa Fluor™ 568 (green) and nuclei were stained with DAPI (blue). Scale is equal the panels and between all zoomed panels, which are taken from the area indicated by the white dotted line

Moreover, uptake studies of RTX-1370S for 45 min at 4 °C demonstrated a decreased uptake compared to the uptake observed at 37 °C for all four cell lines (Fig. 4), similar to what was previously observed for [^111^In]In-FAPI-46 uptake studies. Notably, at 4 °C there seemed to be more membrane-bound RTX-1370S in HT1080-huFAP cells than at 37 °C (Fig. 4C, F), which did not concur with the [^111^In]In-FAPI-46, for which a higher membrane-bound fraction was not found after incubation at 4 °C.

### FAP expression in cell lines and CDX tissue

**Fig. 5:**
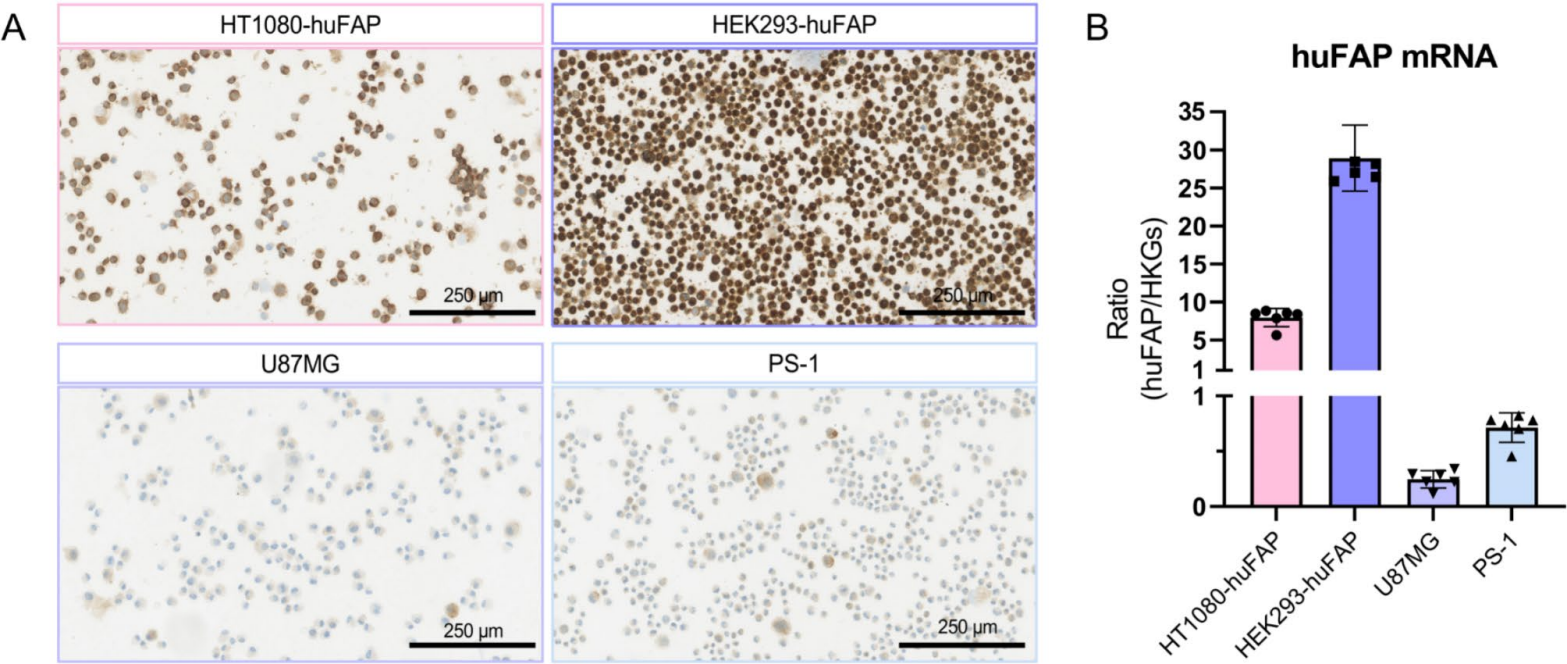
FAP protein and mRNA expression in cell lines. (**A**) Representative images of anti-FAP IHC staining on cytospins of HT1080-huFAP, HEK293-huFAP, U-87 MG, and PS-1 cells and (**B**) mRNA levels expressed as the relative expression ratio between huFAP/HKGs

While the highest radiotracer and fluorescent tracer uptake was observed in the HT1080-huFAP cells, the highest level of huFAP expression was measured in HEK293-huFAP cells, with evidently more intense protein staining (Fig. 5A) and a 3.6-fold higher mRNA level than the HT1080-huFAP cells (8.0 vs. 28.9, respectively) (Fig. 5B). As expected, the U-87 MG and PS-1 cells showed lower huFAP protein (Fig. 5A) and mRNA expression levels (0.24 and 0.71 respectively) (Fig. 5B). Besides, the huFAP expression in PS-1 cells demonstrated to be heterogeneous and seemed to increase with higher passages of the cell (Fig. S4).

**Fig. 6:**
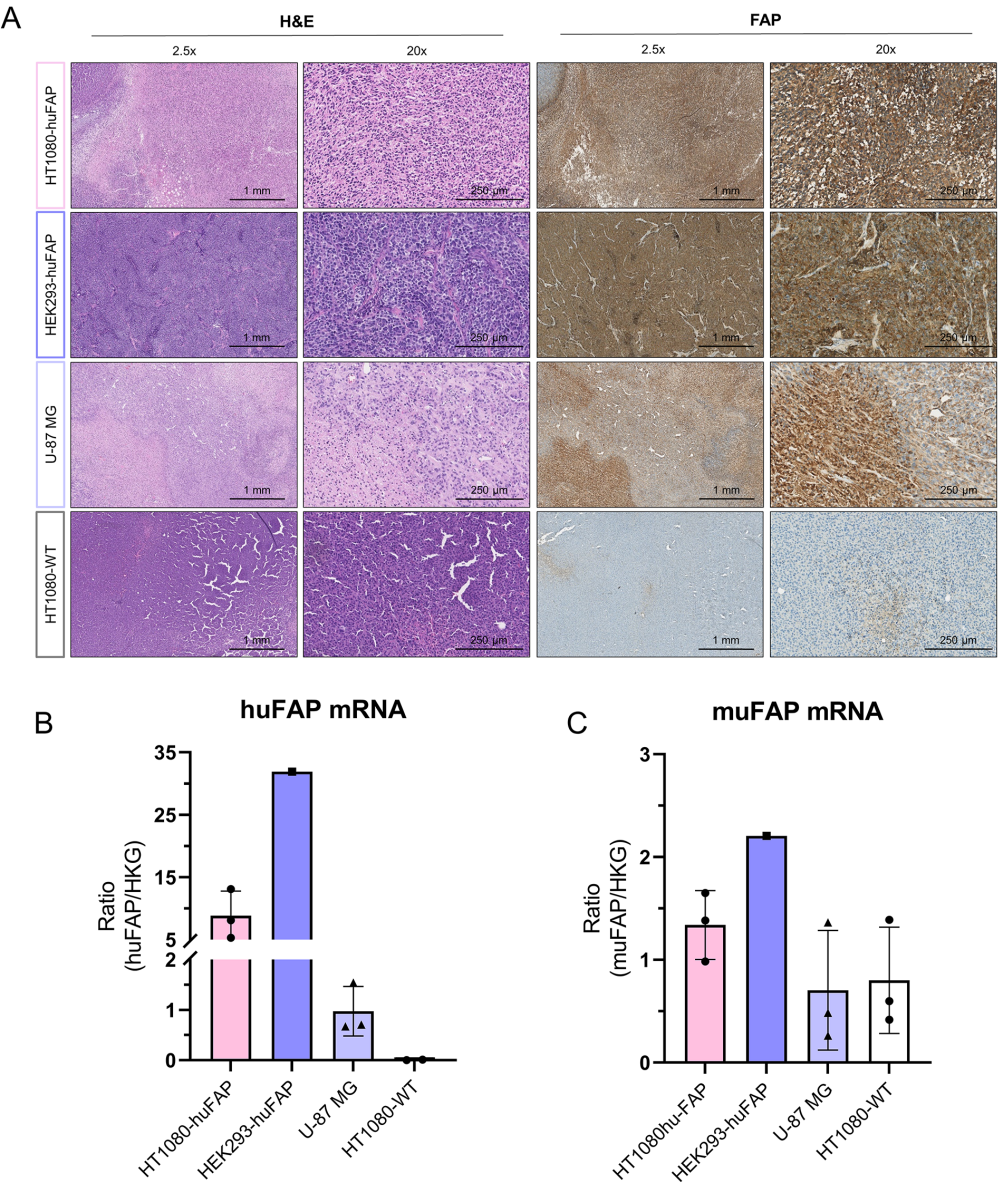
FAP protein and mRNA expression in CDX. (**A**) Representative images of anti-FAP IHC staining on CDX sections of HT1080-huFAP, HEK293-huFAP, and U-87 MG, and mRNA expression levels of (**B**) huFAP and (**C**) muFAP in corresponding CDXs. mRNA levels are expressed as the ratio between huFAP or muFAP and the geometric mean of corresponding human or murine HKGs

Next, it was determined whether the in vivo FAP expression differs from the in vitro expression for the three tumor cell lines, including HT1080-WT as a negative control. The FAP IHC staining revealed a strong FAP expression in the HT1080-huFAP, HEK293-huFAP, and U-87 MG CDXs, while the HT1080-WT CDX showed very few FAP-positive areas (Fig. 6A). The huFAP mRNA expression levels for HT1080-huFAP, HEK293-huFAP and U-87 MG demonstrated an identical in vitro and in vivo pattern (Figs. 5B and 6B). The anti-FAP antibody is known to bind to both huFAP and muFAP, thus cannot distinguish the origin of the FAP. Therefore, the presence of muFAP, as a consequence of murine fibroblast being attracted in vivo, was evaluated by confirming muFAP mRNA expression (Fig. 6C). Notably, we observed that the larger and more necrotic U-87 MG xenografts had higher FAP-expression than the small and slower growing U-87 MG xenografts in our sample set, resulting in both intra- and inter-tumor heterogeneity (Fig. S5). Even though the HT1080-WT demonstrated a similar FAP heterogeneity at mRNA level, it did not show the same FAP heterogeneity at protein level determined by IHC (Fig. 6C, Fig. S5).

### Autoradiography

**Fig. 7:**
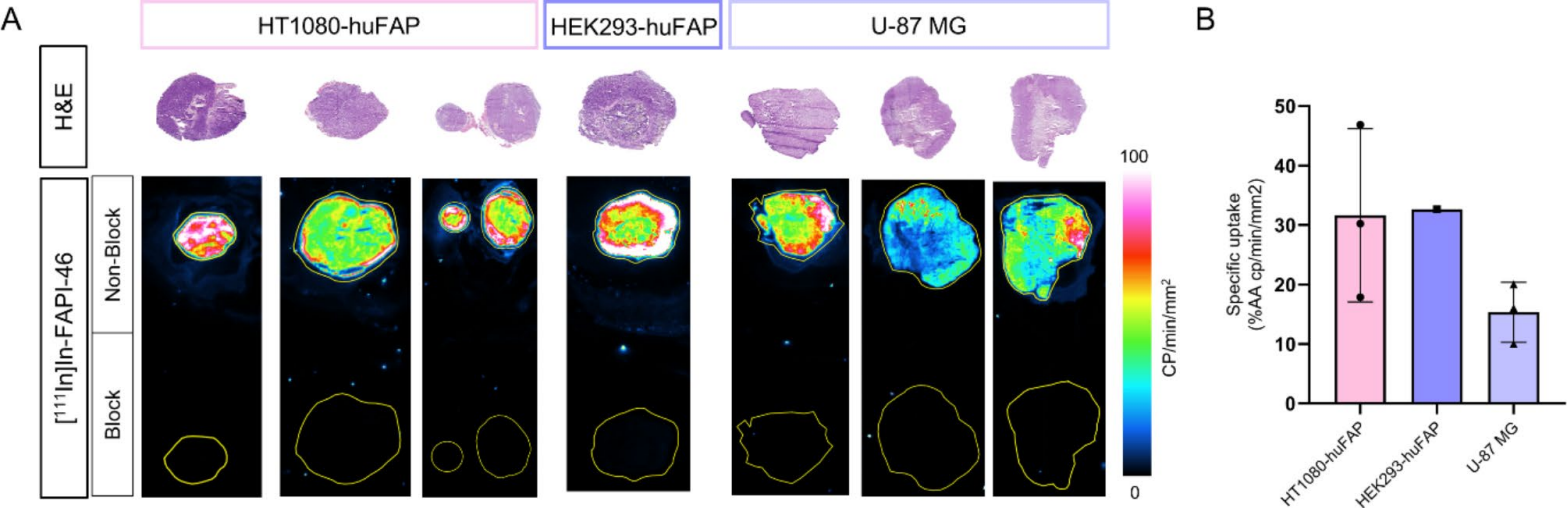
In vitro autoradiography of [^111^In]In-FAPI-46 on CDXs. (**A**) Binding of [^111^In]In-FAPI-46 to CDX material and matching H&E samples. (**B**) Quantification of the [^111^In]In-FAPI-46 binding to CDXs of HT1080-huFAP, HEK293-huFAP, and U-87 MG, calculated for the whole tumor area and expressed as % AA of the counts/min/mm^2^

In line with the uptake studies, the in vitro autoradiography indicated a high specific binding of [^111^In]In-FAPI-46in the CDX of HT1080-huFAP (31.6 ± 14.6% AA) and HEK293-huFAP (32.6% AA), whereas a 2-fold lower binding was observed in the U-87 MG CDX (15.4 ± 5.0% AA) (Fig. 7).

## Discussion

Cancer cell lines transduced to (over-)express human FAP are the most commonly used models to evaluate the potential of novel FAP-targeted radiopharmaceuticals in preclinical studies. However, these models do not accurately mimic the patient situation where the FAP-expression is most often found on CAFs present in the tumor stroma, and it is unclear if these transduced cells mimic the physiological expression and behavior of FAP. To compare tracer behavior in commonly used cell lines, we determined uptake and localization of [^111^In]In-FAPI-46 and RTX-1370S in HT1080-huFAP, HEK293-huFAP, U-87 MG, and an immortalized FAP-expressing fibroblast PS-1. In line with expectations, the highest FAP expression was found in the two transduced cell lines HT1080-huFAP and HEK293-huFAP, resulting in 6–10 fold higher radiotracer uptake in vitro and 2-fold higher radiotracer binding ex vivo compared to the endogenously FAP-expressing U-87 MG and PS-1. Even though HEK293-huFAP cells demonstrated the highest FAP expression, this was not resembled by the uptake of [^111^In]In-FAPI-46, which might indicate that not all of the expressed FAP was available for tracer binding. Alternatively, it might be that the different cell lines have different binding kinetics. The lower levels of FAP expression and [^111^In]In-FAPI-46 uptake observed for PS-1 in vitro and U-87 MG ex vivo are most likely more representative for patients with FAP-positive CAFs infiltrating the tumors (e.g., colon, head and neck cancer, or non-small cell lung cancer), or U-87 MG even in vitro for cancer cells with FAP-expression (e.g., sarcoma, mesothelioma) [15], as it is questionable if the huFAP transduced models with homogeneous over-expression of FAP mimic the physiological expression levels of patients with these cancers.

In addition to the level of uptake, the cellular processing of the radiotracer can influence its performance for FAP-TRT. In line with previously published studies, our in vitro data demonstrated that [^111^In]In-FAPI-46 is rapidly and almost fully internalized [7, 13]. In contrast, confocal microscopy demonstrated that RTX-1370S was at least partly membrane-bound for HEK293-huFAP cells, whereas it is mainly internalized in the other three FAP-expressing cell lines. Unexpectedly, incubating the cells at 4 °C did not alter the ratio between the membrane-bound and internalized [^111^In]In-FAPI-46 for any of the four cell lines. However, a statistically significant decrease in total internalized [^111^In]In-FAPI-46 fraction was observed for HT1080-huFAP and HEK293-huFAP. In contrast, confocal microscopy demonstrated that RTX-1370S remained more membrane-bound at 4 °C in both cell lines. Enzymatic activity, and hereby the internalization rate, was expected to decrease at 4 °C, compared to the physiologically relevant temperature of 37 °C [26]. However, if FAP internalization were a passive process, this would be less impaired at 4 °C than active transport internalization [27]. Additionally, the observed difference in [^111^In]In-FAPI-46 and RTX-1370S localization could be caused by the Alexa Fluor™ 568 group that contributes to the larger size of the fluorescent tracer, and that can lead to a higher hydrophilicity of RTX-1370S over FAPI-46. Thus the observed differences could also be the result of differences between the tracers. This has also been observed in a study by Millul et al., who compared multiple FAP-targeted radiotracers using two huFAP transduced cell lines. The study demonstrated complete internalization of FAPI-46, while FAP-2286 remained membrane-bound in both cell lines [13]. Similar to our study, the researchers removed the surface-bound radiotracer with an acid glycine buffer (pH 2.8), which is commonly used to interrupt target-ligand interactions, assuming that this interaction is pH dependent [28]. However, studies have shown that using an acid wash to separate the membrane-bound fraction is not always effective and reliable, and is dependent on the protein’s sensitivity to pH and the type of target-ligand interaction [29]. It has been confirmed that binding affinity of FAP for a ligand can be pH dependent [30], but it is unclear at which pH the interaction between a FAP-targeted small molecule and FAP could be disrupted. This target-ligand pH dependency could be different for the cyclic peptide FAP-2286 and FAPI-46, and further research could help identify if this acid wash is the optimal strategy to separate membrane-bound small molecule FAP inhibitors from FAP. Thus, based on our data it remains inconclusive if the observed differences in localization of [^111^In]In-FAPI-46 and RTX-1370S are due to differences in tracer behavior, the difference in the tracer concentration (1 nm and 10 nm, respectively), or due to suboptimal separation of membrane-bound radiotracer. Nevertheless, confocal microscopy did demonstrate a different localization pattern for RTX-1370S in HT1080-huFAP and HEK293-huFAP cells, indicating that tracer behavior is impacted by the model.

There are multiple explanations possible for the observed differences between HT1080-huFAP and HEK293-huFAP. Although both have been transduced to express the huFAP gene, the transductions were performed using different vectors and protocols, which could introduce differences in the expression levels of FAP. Additionally, the huFAP introduced in the HEK293 cell line is fused with GFP, and this fusion protein could alter the internalization kinetics of FAP. The impact of the GFP fusing affecting the FAP internalization is also supported by the observation of a high GFP signal around the nucleus, and almost 30% of the FAP-GFP not co-localizing with RTX-1370S. Thus, it seems that not all FAP is available for tracer binding, potentially due to FAP accumulation in the endoplasmic reticulum. This is in line with our data demonstrating the highest levels of FAP protein and mRNA expression in HEK-293-huFAP cells, which did not result in the highest [^111^In]In-FAPI-46 uptake. Thus, our data demonstrates various differences in FAP expression, internalization, and processing. Because these mechanisms are currently not fully understood, it is difficult to select the model with the highest translational value.

During our study, we observed that the FAP expression of PS-1 cells increased with higher passage numbers. This trend was observed with consecutive uptake studies, hence the higher SD in our results compared to that of the other cell lines. By IHC it was determined that FAP-expression was higher in passage 27 than in passage 22. The PS-1 cell line is hTERT immortalized, and a study by Park et al. found that hTERT expression can increase FAP expression by interacting with a transcription factor that is involved in regulating FAP expression (i.e., EGR1) [31]. Thus, endogenous expression should be interpreted carefully in the case of (hTERT) immortalization of primary cells, and ideally FAP expression prior to immortalization should be determined to establish if FAP expression was impacted by hTERT introduction.

Almost no studies described isolated primary human CAFs as a model to evaluate FAP-targeted radiotracers. Although these would have the highest translational value, it is practically challenging and difficult to reproduce. However, for FAP-TRT studies, a monoculture of CAFs would still not be adequate to study the complex aspects of indirect radiation of cancer cells after radiotracer binding to the CAFs. Thus, more advanced models such as co-cultures or 3D organoid models are necessary to determine and compare the FAP-TRT efficacy of radiotracers in vitro. Although these models are more complex, their value is extensively being researched [32–34]. Indirect co-cultures are the most user-friendly alternative to mono-cultures, as it allows separation of the two cell types while including the effects of the secretions and cross-talk between CAFs and the cancer cells. Nevertheless, the distance between the cells is still not representative of TRT in the clinical situation, and thus suboptimal to evaluate the efficacy of FAP-TRT. Alternatively, direct 2D co-cultures consisting of CAFs and cancer cells mimicking a variation of cancer types are extensively being researched to study the interaction between CAFs and cancer cells [33, 35, 36]; however, to our knowledge, no studies have been published describing TRT in these direct co-culture models to date. More complex 3D spheroid and organoid models are also becoming an attractive alternative to 2D cell culture. For example, Schuth et al. developed a personalized primary pancreatic ductal adenocarcinoma co-culture model to study the impact of CAFs on cancer cell response to chemotherapy [32]. However, studies describing TRT in such advanced models are lacking, implying that using radiotracers in a 3D organoid model is complicated.

For the evaluation of novel radiotracers, in vivo data remains essential to determine therapeutic efficacy and tumor-to-background ratios. The U-87 MG cells are an attractive model for FAP-TRT research because these cells stimulate attraction of murine fibroblasts in vivo. Thus, the level of FAP expression in this model depends on the attraction of enough murine fibroblasts, resulting in a heterogenic FAP expression. Despite this lower and heterogenic FAP-expression, we only observed a 2-fold lower binding of FAPI-46 to U-87 MG xenografts compared to HT1080-huFAP and HEK293-huFAP xenografts with in vitro autoradiography, whereas an almost 10-fold higher in vitro cell uptake was observed. One limitation of this research was the availability of fresh frozen HEK293-huFAP xenografts, hence no evaluation of the heterogeneity of binding in this CDX model could be performed. In a future study it would be valuable to include a large sample set with a variety of huFAP transduced cell lines to evaluate their differencesin vitro and in vivo, and to determine the impact on FAP-targeted radiotracer evaluation in more detail. Nevertheless, U-87 MG CDXs seem more suitable for in vivo TRT-efficacy studies, likely with a higher translational value compared to a xenograft established with a FAP over-expressing cell line. Next to U-87 MG, other cell lines have been used to create a FAP-positive CDX based on attracting murine fibroblasts, including the pancreatic cancer cell lines Panc-1 and PDAC299 [37–39]. PDAC299, for example, demonstrates high histopathological similarities with the stroma-dense pancreatic ductal adenocarcinomas in patients. Even though these xenografts can demonstrate resemblance to a patient’s tumor, the FAP expression in healthy organs remains different between mice and man, especially due to the higher levels of soluble FAP in mice [40]. Even more advanced in vivo models are available, such as the genetically engineered KPC mouse model for PDAC [41]. Although this model resembles patient histology more closely, these models are more labor-intensive, expensive, and not available for all tumor types. Studies using any in vivo model based on attraction of muFAP-expressing CAFs should carefully consider the radiotracer’s affinity for huFAP and muFAP. FAPI-46 demonstrated high and comparable affinity for both muFAP and huFAP, but FAP-2286, for example, does demonstrate a notable difference in affinity between the two isoforms [15, 21]. All in all CDXs and other in vivo models remain an approximation of the clinical situation, but with increased understanding of the FAP-expressing models, the most representative model can be selected.

## Conclusions

In conclusion, it is critical to select a preclinical model that is relevant to the proposed research question, especially for FAP-targeted theranostics. Our data indicates that understanding and characterizing in vitro and in vivo FAP-expressing models can help to select the right system with the highest translational value, ultimately accelerating the clinical implementation of novel FAP-targeted radiotracers.

## Electronic supplementary material

Below is the link to the electronic supplementary material.

Supplementary Material 1

## Data Availability

All available data are described in the manuscript or are available in the supplementary materials. Additional information can be obtained from the corresponding author on reasonable request.

## Abbreviations

% AA: Percentage Added Activity
CAF: Cancer-Associated Fibroblast
CDX: Cell Line Derived Xenograft
FAP: Fibroblast Activation Protein
H&E: Hematoxylin and Eosin
hTERT: human Telomerase Reverse Transcriptase
huFAP: human FAP
IHC: Immunohistochemistry
muFAP: murine FAP
RT-qPCR: Reverse Transcription Quantitative Polymerase Chain Reaction
TRT: Targeted Radionuclide Therapy
RT: Room Temperature
