# Supplementary material for: In vitro and ex vivo evaluation of preclinical models for FAP-targeted theranostics: differences and relevance for radiotracer evaluation
Source: EJNMMI Res. 2024 Dec 24;14:125. doi: 10.1186/s13550-024-01191-6 (PMC11668701; doi:10.1186/s13550-024-01191-6)
Supplement: Supplementary file 1 — Supplementary Material 1 [file 13550_2024_1191_MOESM1_ESM.docx]

**Supplementary information: In vitro and ex vivo evaluation of preclinical models for FAP-targeted theranostics: Differences and relevance for radiotracer evaluation**

Circe D. van der Heide^1^, Joana D. Campeiro^1^, Eline A.M. Ruigrok^1^, Lilian van den Brink^1^, Shashikanth Ponnala^2^, Shawn M. Hillier^2^, Simone U. Dalm^1^.

^1^*Department of Radiology & Nuclear Medicine, Erasmus MC University Medical Centre Rotterdam, Rotterdam, 3015 GD, The Netherlands.*

^2^*Ratio Therapeutics, Inc., Boston, USA*

Corresponding author: Simone U. Dalm – [s.dalm@erasmusmc.nl](mailto:s.dalm@erasmusmc.nl)

**Supplementary materials and methods**

*General* *liquid chromatography–mass spectrometry (LCMS) Conditions*

Mass spectra (LCMS) analysis was performed using Waters Auto pure Mass Spectrometer using X-Bridge premier BHE C18 3.5 µM (4.6 X 50 mm) column. Accurate m/z are reported for the molecular ion [M+H]+.

*Chemistry of RTX-1370S*

**tert-butyl N2-(N2-(((9H-fluoren-9-yl)methoxy)carbonyl)-N6-(tert-butoxycarbonyl)-L-lysyl)-N6-(2-(4-isobutylphenyl)acetyl)-L-lysinate** **(3)**: A solution of **1** (4.96g, 10 mmol), **2** (3.76g, 10mmol), EDC.HCl (2.29 g, 1.2 mmol), and HOBt (1.862g, 1.2 mmol) in DCM (100 mL) was stirred for 10 min and DIEA (3 equiv.) was added slowly at 0 ^o^C. The stirred reaction mixture was monitored by LC-MS for 4 h at room temperature. The mixture was diluted with DCM and the organic layer was washed sequentially with water and brine, dried over MgSO4, filtered, and the volatiles were removed under reduced pressure to afford the crude product, which was purified by combi-flash silica gel chromatography (DCM and EtOAc eluant) to obtain the pure **3** 1(5.78g, 70% yield). LCMS: calcd for C_48_H_66_N_4_O_8_, 826.49; observed m/z = 827.6 [M+H]^+^.

**tert-butyl N2-(N6-(tert-butoxycarbonyl)-L-lysyl)-N6-(2-(4-isobutylphenyl)acetyl)-L-lysinate (4):** To a solution of **3** (3.46g, 4.2mmol) in DCM (20 mL) was added diethylamine (6.51 ml, 62.59 mmol) at ambient temperature. Full conversion was detected by LCMS after 45 min. The solvents were evaporated under reduced pressure and the residue was dissolved in DCM purified on a CombiFlash apparatus (220 g silica gel column, gradient 0 to 50% MeOH/DCM over 100 minutes). The desired fractions were combined and evaporated to produce target material (1.34g, 54%) as amorphous colorless solid **4**. LCMS: calcd for C_33_H_56_N_4_O_6_, 604.42; observed m/z = 605.3 [M+H]^+^.

**tert-butyl (18S,20R)-18-(4-((tert-butoxycarbonyl)amino)butyl)-1-(9H-fluoren-9-yl)-20-(5-(2-(4-isobutylphenyl)acetamido)pentyl)-3,16,19-trioxo-2,7,10,13-tetraoxa-4,17-diazahenicosan-21-oate (6):** DIEA (1.34 ml, 1.72 mmol) was added to a stirred solution of **4** (2.12g, 3.51 mmol), **5** (1.554, 3.51 mmol) and HATU (1.47 g, 3.86 mmol) in DMF (10 ml) at ambient temperature. The reaction mixture was stirred at room temperature for 14 h, at which time full conversion was detected by LCMS. The mixture was diluted with EtOAc, washed with water (x2) and brine, dried over anhydrous MgSO_4_ and filtered. The filtrate was evaporated to obtain target material **6** (2.88g, 80%) as a colorless amorphous solid. LCMS: calcd for C_58_H_84_N_4_O_12_, 1028.61; observed m/z = 1029.8 [M+H]^+^.

**tert-butyl(14S,16R)-1-amino-14-(4-((tert-butoxycarbonyl)amino)butyl)-16-(5-(2-(4-isobutylphenyl)acetamido)pentyl)-12,15-dioxo-3,6,9-trioxa-13-azaheptadecan-17-oate (Intermediate-A):** To a solution of **6** (2.88g, 2.80mmol) in DCM (20 ml) was added diethylamine (4.34 mL) at ambient temperature. Full conversion was detected by LCMS after 45 min. The solvents were evaporated under reduced pressure and co distilled with ACN (20mL x3), dried under vacuum. The crude product was used without any further purification in the next step. LCMS: calcd for C_43_H_74_N_4_O_10_, 806.54; observed m/z = 807.4 [M+H]^+^.

**[2-((S)-2-cyano-pyrrolidin-1-yl)-2-oxo-ethyl]-carbamic acid tert-butyl ester (8)**: To a solution of **7** (1 g, 7.5 mmol) in DCM (35 ml) was added DIEA (3.9 ml, 22.5 mmol) and Boc-Gly-OSu (2.26 g, 8.3 mmol). The reaction mixture was stirred at ambient temperature for 14 h. The solvents were evaporated, and the residue was dissolved in DCM, washed with water, dried over anhydrous MgSO_4_ and filtered. The filtrate was concentrated to 20% of the initial volume and loaded onto a CombiFlash™ apparatus and eluted (gradient 0 to 10% MeOH/DCM). The desired fractions were combined and evaporated to obtain target product (1.1 g, 58%) as an amorphous solid. LCMS: calcd for C_12_H_19_N_3_O_3_, 253.14; observed m/z = 254.4 [M+H]^+^.

**(S)-1-(2-amino-acetyl)-pyrrolidine-2-carbonitrile (9)**: p-Toluenesulfonic acid monohydrate (1.25 g, 6.6 mmol) was added to a stirred solution of **8** (1.1 g, 4.4 mmol) in acetonitrile (20 ml) and the mixture was stirred at ambient temperature for 14 h. The solvent was removed at reduced pressure to obtain the tosylate salt of the target product (1.8 g, yield exceeded theoretical). The compound was sufficiently pure to carry through to the next chemical transformation. LCMS: calcd for C_7_H_11_N_3_O, 153.3; observed m/z = 154.6 [M+H]^+^.

**(S)-N-(2-(2-cyanopyrrolidin-1-yl)-2-oxoethyl)-7-hydroxyquinoline-4-carboxamide (11):** A solution of **10** (0.306g, 1.62 mmol), HOBt (0.221g, 1.62 mmol), and TBTU (521 mg, 1.62 mmol) in DMF (15 mL) was stirred at room temperature for 5 min. A solution of **9** (0.578g, 1.78 mmol) and DIEA (0.74 mL, 4.86 mmol) in DMF (5 mL) was added to the activated ester solution and the resulting mixture was stirred at room temperature for 2 h while the reaction progress was monitored by LCMS. Upon full conversion, the reaction mixture was concentrated under reduced pressure, dissolved in a small amount of dichloromethane, and filtered. The filtrate was evaporated, dissolved in DCM, loaded onto a CombiFlash™ silica gel column, and purified (gradient 0->20% MeOH/DCM) to obtain **11** (422 mg, 80% yield) as an off-white solid. LCMS: calcd for C_17_H_16_N_4_O_3_, 324.12; observed m/z = 325.2 [M+H]^+^.

**(S)-tert-butyl 2-((4-((2-(2-cyanopyrrolidin-1-yl)-2-oxoethyl) carbamoyl)quinolin-7-yl)oxy)acetate (12):** A flask containing **11** (0.407g, 1.25 mmol), 2-tert-butyl glycolate (0.249g, 1.88 mmol), and triphenylphosphine (395 mg, 1.51 mmol) in DMF (15 mL) was chilled in an ice water bath. Di-isopropyl azodicarboxylate (300 uL, 1.51 mmol) was added dropwise to the chilled reaction mixture. The ice water bath was removed, and the resulting solution was stirred at room temperature and monitored by LCMS. Upon completion, the solvent was removed under reduced pressure and the residue was dissolved in DCM, loaded onto a CombiFlash™ silica gel column (gradient 0->10% MeOH/DCM) and purified to obtain **12** (404 mg, 61% yield) as a glassy solid. LCMS: calcd for C_23_H_26_N_4_O_5_, 438.19; observed m/z = 439.1 [M+H]^+^.

{**4-[2-((S)-2-Cyano-pyrrolidin-1-yl)-2-oxo-ethylcarbamoyl]-quinolin-7-yloxy}-acetic acid** **(Intermediate-B):** To a solution of **12** (0.141g, 0.322 mmol) in DCM (1.5 ml) was added TFA (1.51 ml,19.64 mmol) dropwise at 0 °C. The temperature of the stirred reaction mixture was allowed to rise to room temperature over the course of 1 h, at which time full conversion was detected by LCMS. The solvent was evaporated at room temperature (rt) and residue was co-evaporated with toluene (x3) at 40 °C. The crude Intermediate**-B** was sufficiently pure to use for subsequent transformations. LCMS: calcd for C_19_H_18_N_4_O_5_, 382.13; observed m/z = 383.1 [M+H]^+^.

**tert-butyl (17S,19R)-17-(4-((tert-butoxycarbonyl)amino)butyl)-1-((4-((2-((S)-2-cyanopyrrolidin-1-yl)-2-oxoethyl)carbamoyl)quinolin-7-yl)oxy)-19-(5-(2-(4-isobutylphenyl)acetamido)pentyl)-2,15,18-trioxo-6,9,12-trioxa-3,16-diazaicosan-20-oate (13):** DIEA (1.64 ml, 9.43 mmol) was added to a stirred mixture of **Intermediate-B** (1.57 mmol), **Intermediate-A** (1.39 g, 1.73 mmol) and HATU (717 mg, 1.89 mmol) in DMF (10 ml). The stirred mixture was held at room temperature for 30 minutes, when full conversion was detected by LCMS. The mixture was diluted with EtOAc, washed with water and brine, and the collected aqueous phase was extracted with EtOAc. The combined organic extracts were washed with brine, dried over anhydrous MgSO4, filtered, and evaporated under reduced pressure. The residue was dissolved in DCM, loaded onto a CombiFlash silica gel column (220 g, gradient 0->30% MeOH/DCM in 60 minutes) and purified. The desired fractions were combined and evaporated to produce target material (1.17g, 58%) as glassy solid. LCMS: calcd for C_62_H_90_N_8_O_14_, 1170.66; observed m/z = 1172.6 [M+H]^+^.

**(17S,19R)-17-(4-aminobutyl)-1-((4-((2-((S)-2-cyanopyrrolidin-1-yl)-2-oxoethyl)carbamoyl)quinolin-7-yl)oxy)-19-(5-(2-(4-isobutylphenyl)acetamido)pentyl)-2,15,18-trioxo-6,9,12-trioxa-3,16-diazaicosan-20-oic acid (14):** To a solution of **13** (0.072g, 0.071 mmol) in anhydrous DCM (1 ml) was added anhydrous trifluoroacetic acid (1 ml) dropwise at 0 °C. The temperature of the reaction mixture was allowed to rise to ambient and stirring was continued for 90 minutes, at which time full conversion was detected by LCMS. The solvent was evaporated at room temperature and residue was co-evaporated with toluene. The compound so obtained (**14**) compound was used immediately in the next chemical transformation. LCMS: calcd for C_53_H_74_N_8_O_12_, 1015.56; observed m/z = 1016.6 [M+H]^+^.

**(6-(2-carboxy-4-(((S)-17-(((S)-1-carboxy-5-(2-(4-isobutylphenyl)acetamido)pentyl)carbamoyl)-1-((4-((2-((S)-2-cyanopyrrolidin-1-yl)-2-oxoethyl)carbamoyl)quinolin-7-yl)oxy)-2,15-dioxo-6,9,12-trioxa-3,16-diazahenicosan-21-yl)carbamoyl)phenyl)-2,2,10,10-tetramethyl-10,11-dihydro-2H-pyrano[3,2-g:5,6-g']diquinoline-1-ium-4,8-diyl)dimethanesulfonate (RTX-1370S):** To the solution of **AF-568** (**15**) (0.070 g, 0.067 mmol) in DMSO (1 mL) was added to a solution of **14** (0.068 g, 0.045 mmol) in DMSO (1 mL). N,N-Diisopropylethylamine (0.070 g, 0.067 mmol) was added at room temperature, and the mixture was allowed to stir for 16h. Full conversion was detected by LCMS. After the reaction completion, the volatiles were removed, and the residue was purified by HPLC to yield RTX-1370S (15 mg, 14%) as a green solid. LCMS: calcd for C_85_H_100_N_11_O_22_S_2_, 1691.93; observed m/z = 1694.3 [M+3H]^+^.

*Transduction of HEK293 cells*

Transduction of the human embryonic kidney cells (HEK293) cells was performed with lentiviral vector construct pLV[Exp]-Puro-EF1A>hFAP[NM_004460.5](ns):3xGGGGS:sfGFP (VectorBuilder, Chicago, USA). For selection of the transduced cells, the cells were maintained in a 1.5 µg/ml Puromycin-containing growth medium. After single-cell clones were isolated and expanded, huFAP expression was confirmed by both RT-qPCR and flow cytometry using an anti-human fibroblast activation protein (FAP) antibody (Bio-techne, Minneapolis, USA).

*Immunohistochemistry (IHC)*

Hematoxylin and eosin (H&E) staining was performed using the fully automated HE600 (Ventana) according to the manufacturer’s instructions. The anti-FAP polyclonal antibody EPR20021 (Abcam, Cambridge, UK) was used in 1/50 dilution, and tissues and cells were stained for FAP using optiview (OV) (#760-700, Ventana). The anti-FAP DAB staining of the cytospins was performed by automated IHC using the Ventana Benchmark ULTRA (Ventana Medical Systems). After deparaffinization and heat-induced antigen retrieval with CC1 (#950-500, Ventana) for 8 min, the cytospins were incubated with the anti-FAP antibody for 32 min at 37˚C. The FAP staining on xenograft tissue was performed by automated immunofluorescence using the Ventana Benchmark Discovery ULTRA (Ventana Medical Systems Inc.). The formalin-fixed paraffin-embedded sections were prebaked at 60°C followed by deparaffinization and heat-induced antigen retrieval with CC1 (#950-224, Ventana) for 32 min. Next anti-FAP was incubated for 32 min at 37°C followed by omnimap anti-rabbit HRP (#760-4311, Ventana) and detection with chromomap DAB kit (760-159, Ventana). After incubation, all samples were subjected to optiview detection and hematoxylin II counter staining and bluing according to the manufacturer’s instructions.

*RNA isolation*

To isolate RNA, 800 µL TRIzol^®^ reagent (Invitrogen, Carlsbad CA, USA) was added to 1×10^6^ cells or 1 mL to 30 mg of tumor tissue, and collected in Eppendorf tubes. Hereafter, 200 µL chloroform was added to the TRIzol^®^ reagent and the samples were centrifuged at 12,000 *xg* for 15 min at 4°C. To obtain the isolated RNA, the aqueous phase was collected and the RNA was precipitated using 0.5 mL isopropanol followed by centrifuging at 16,000 x*g* for 10 min at 4°C. Subsequently, the pellet containing the RNA was washed with ice cold 75% ethanol and centrifuged at 12,000 *xg* for 10 min at 4°C. The supernatant was discarded and the pellet was dissolved in 30 µL of RNase-free water.

*Tumor inoculation details for generation of CDX material*

Female Nu/J (inbred) athymic nude mice (Jackson Laboratory, Bar Harbor, ME) 8-10 weeks old were subcutaneously inoculated with 100 µL containing 5×10^6^ U-87 MG cells, diluted 1:1 in 1/2 Matrigel and 1/2 DMEM. The xenografts derived from human (hu)FAP transduced HEK293 cells (HEK293-huFAP) were generated by subcutaneous inoculation of 5×10^6^ cells, diluted 1:1 in 1/2 Matrigel and 1/2 DMEM, on the right flank of 8-10 week old female NCr-Foxn1^nu^ (Taconic Biosciences, Germantown, NY). Tumor tissue was collected at a volume of 125 – 500 mm^3^. Tumors were either immediately snap-frozen using hexane on dry ice, or fixed in 10% buffered formalin for 24 h and then transferred to 70% ethanol for preservation and subsequently embedded in paraffin.

**Supplementary Tables and Figures**

**Scheme 1** Chemical synthesis of RTX-1370S

**Supplementary Table 1.** Primer sequences of the forward (Forw.) and reverse (Rev) primer for RT-qPCR studies

| **Gene** | **Primer** | **Sequence (5’ 🡪 3’)** |
| --- | --- | --- |
| **Human FAP** | Forw. | CAAAGGCTGGAGCTAAGAATCC |
|  | Rev. | ACTGCAAACATACTCGTTCATCA |
| **Murine FAP** | Forw. | GACGGGGGACTGACTTTCTG |
|  | Rev. | CTAACCTCCTGAGCCCTCCT |
| **Human ACTB** | Forw. | CTTCGCGGGCGACGAT |
|  | Rev. | CCACATAGGAATCCTTCTGACC |
| **Human HPRT** | Forw. | CACTGGCAAAACAATGCAGACT |
|  | Rev. | GTCTGGCTTATATCCAACACTTCGT |
| **Human GUSB** | Forw. | GAGCAAGACAGTGGGCTGG |
|  | Rev. | CCATTCGCCACGACTTTGTT |
| **Murine HPRT1** | Forw. | TCCTCCTCAGACCGCTTTT |
|  | Rev. | CCTGGTTCATCATCGCTAATC |
| **Murine TBP** | Forw. | GGAGAATCATGGACCAGAACA |
|  | Rev. | GATGGGAATTCCAGGAGTCA |

FAP = Fibroblast activation protein, ACTB = beta actin, HPRT = hypoxanthine guanine phosphoribosyltransferase, GUSB = beta glucuronidase, TBP= TATA-box binding protein

**
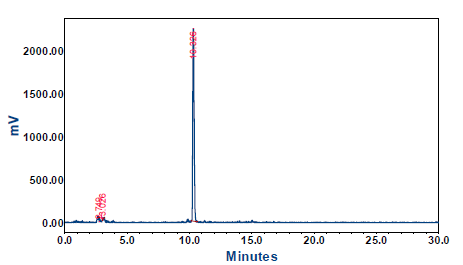
**

**Supplementary Fig. S1. Radio-** **high-performance liquid chromatography (HPLC) chromatogram [^111^In]In-FAPI-46.** Radio-HPLC demonstrating a radiochemical purity of 95.6% for [^111^In]In-FAPI-46.


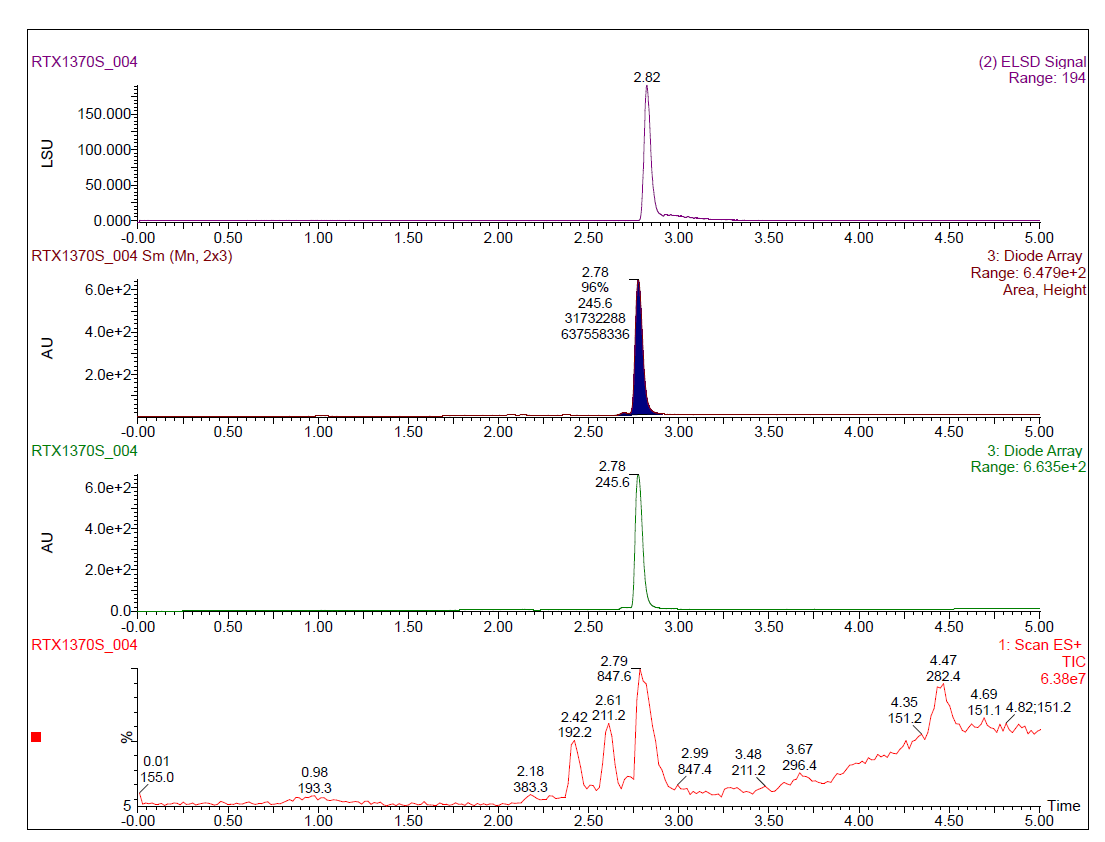


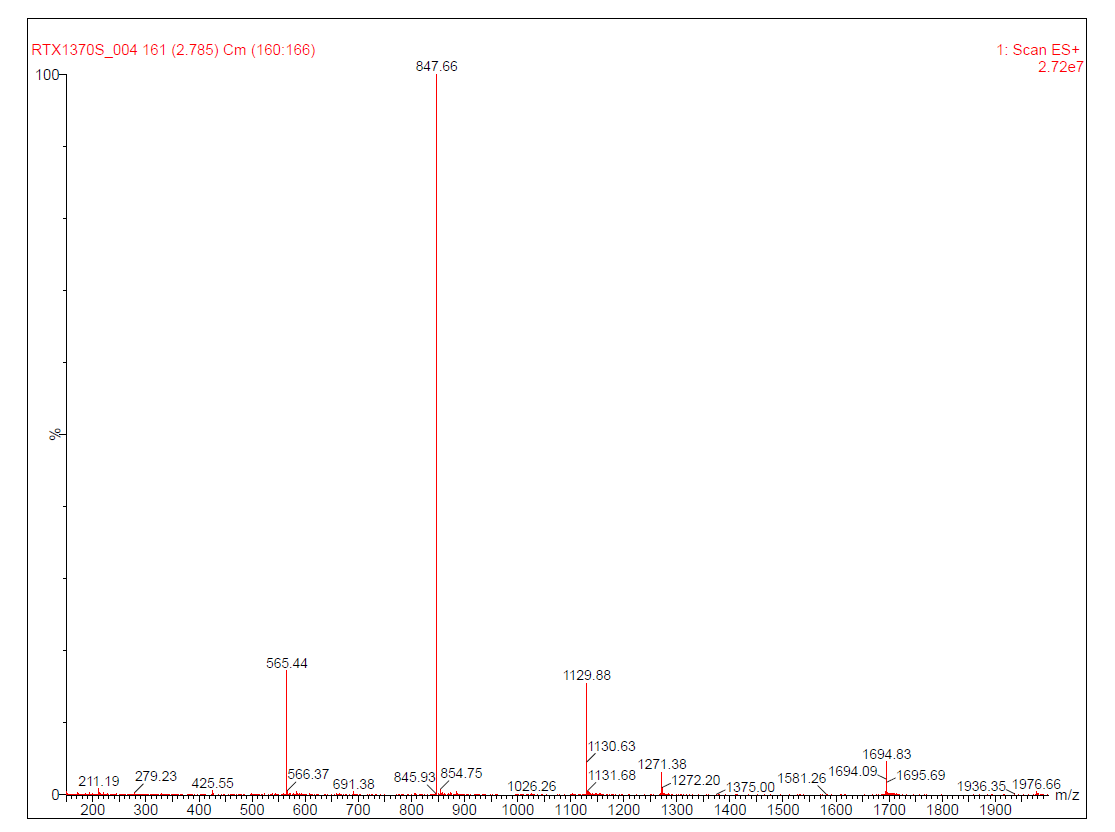


**Supplementray Fig. S2. Mass spectrum analyses of RTX-1370S by LCMS.**


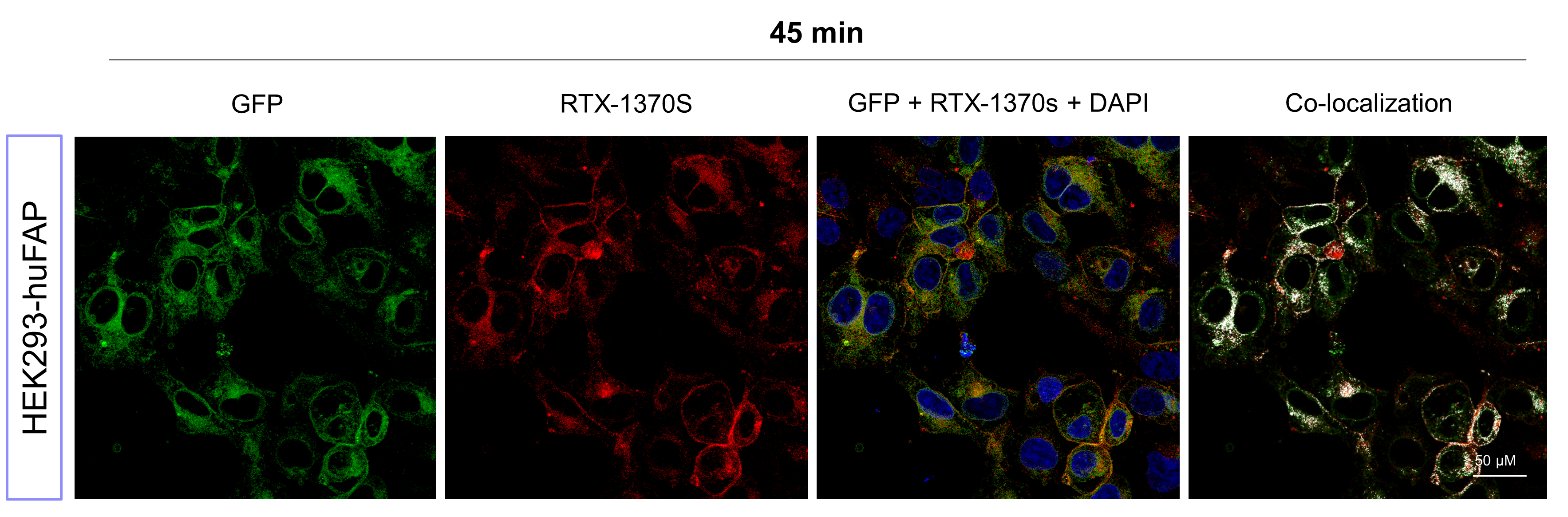


**Supplementary Fig. S3. RTX-1370S uptake at 37°C on HEK293-huFAP.** Confocal microscopy of HEK293-huFAP, incubated with 10 nM of RTX-1370S for 45 min at 37°C. HEK293-huFAP cells express huFAP-GFP fusion protein (green), RTX-1370S was conjugated to an Alexa Fluor™ 568 (red), and nuclei were stained with DAPI (blue), in the last panel co-localization of GFP and RTX-1370S is highlighted (white)

**
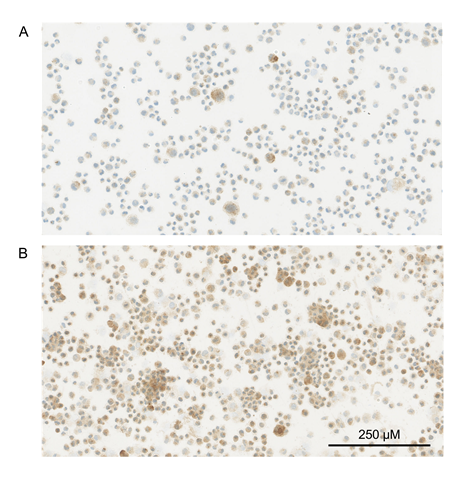
**

**Supplementary Fig. S4.** FAP expression in PS-1 cells over time**.** Representative images of anti-FAP IHC on PS-1 cells cultured at (A) passage 22 and (B) passage 27. The magnification is equal in both images


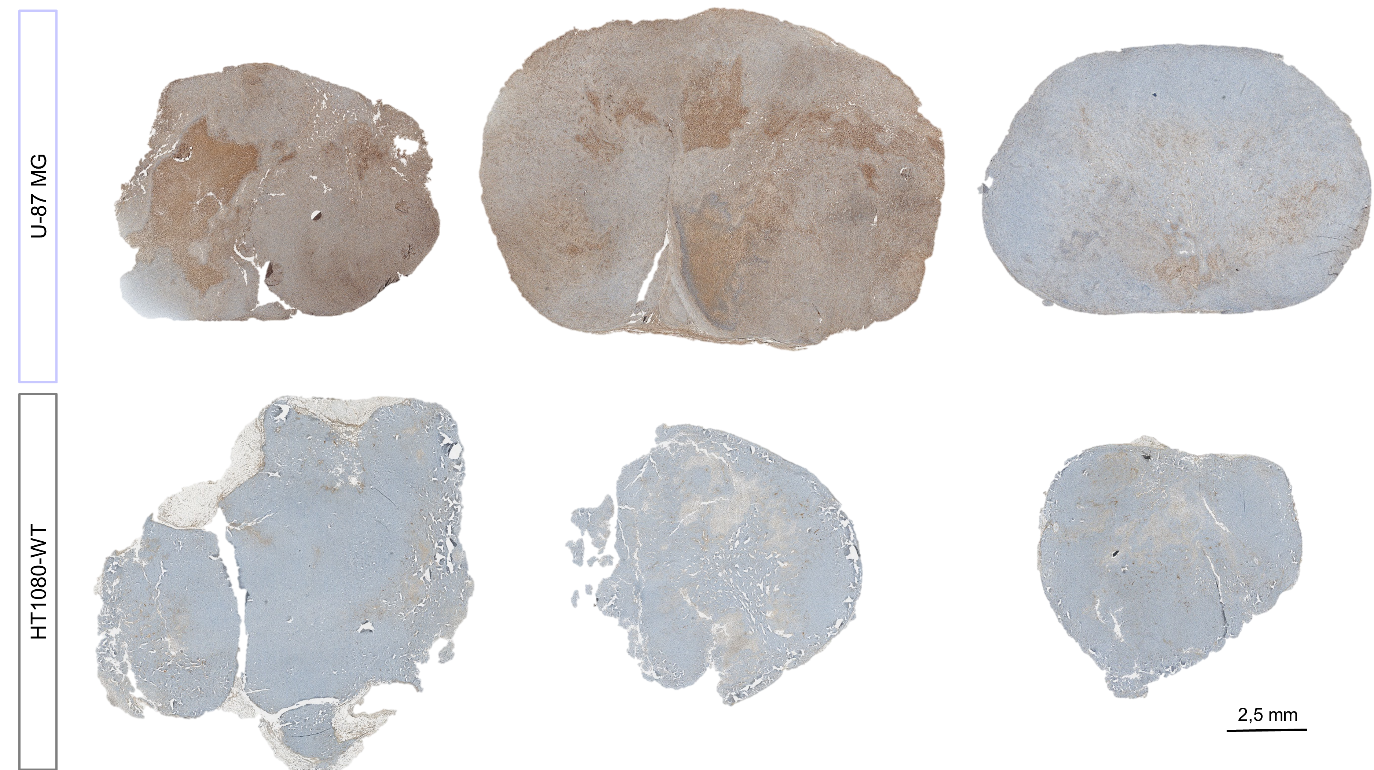


**Supplementary Fig. S5.** FAP expression in U-87 MG and HT1080-huFAP xenografted tumors**.** Anti-FAP IHC on U-87 MG and HT1080-WT xenograft tumors. The magnification is equal in all images
